# Awareness and utilization of community clinic services among women in rural areas in Bangladesh: A cross-sectional study

**DOI:** 10.1371/journal.pone.0187303

**Published:** 2017-10-27

**Authors:** Sanni Yaya, Ghose Bishwajit, Michael Ekholuenetale, Vaibhav Shah

**Affiliations:** 1 School of International Development and Global Studies, University of Ottawa, Ottawa, Ontario, Canada; 2 Institute of Nutrition and Food Science, University of Dhaka, Dhaka, Bangladesh; 3 Women's Health and Action Research Centre, Benin City, Nigeria; 4 Interdisciplinary School Health Sciences, University of Ottawa, Ottawa, Ontario, Canada; Centre for Injury Prevention and Research, Bangladesh (CIPRB) & Örebro University, Sweden, BANGLADESH

## Abstract

**Background:**

In recent years, Bangladesh government has accomplished the ambitious project of establishing hospitals 18,000 Community Health Clinics in sub-districts across the country. Operating under the affiliation of the government hospitals, these community health clinics aim to provide free healthcare services and to increase health-awareness among the extreme poor communities in the rural areas. However, a great proportion of the people are still not well aware of the services offered by the community health clinics. Thus, it is imperative to identify the factors of awareness regarding the community clinics. Research-based evidence is necessary to improve the efficacy and service coverage of community clinics among key population.

**Methods:**

Cross-sectional data of size 11,673 women aged 15 to 49 years living in rural settings across seven divisions were extracted from the latest Bangladesh Demographic and Health Survey 2014. The main outcome measures of our study were awareness and utilization of Community Clinic Services (CCs). Descriptive statistics were used to present the baseline socio-demographic and economic characteristics; Chi-square test and logistic regression were performed to identify the factors associated with awareness of community clinics.

**Results:**

About one-third (36.7%) of the women were aware of community clinics. Geographical location, level of education, household wealth status and frequency of reading newspaper were found to be significantly associated with awareness about community clinic services. Services reported to be obtained in the community clinics include family planning, immunization, tetanus, antenatal care, vitamin A, and health care for children and child growth monitoring. In the multivariate logistic regression, the odds of awareness among participants with primary education [p<0.001, AOR = 1.255, 95%CI = 1.107–1.357], secondary qualification [p<0.001, AOR = 1.370, 95%CI = 1.242–1.510] and tertiary [p<0.001, AOR = 1.526, 95%CI = 1.286–1.809] had approximately 23%, 37% and 53% respectively higher odds of awareness when compared to those with no formal education. Compared to the women living in richest households, odds of awareness were approximately 12.5%, 12.8%, 4.5% and 22.4% respectively higher among women reported in poorer, middle, richer and richest household wealth status when compared to poorest wealth status.

**Conclusion:**

Our findings suggested that policies enhancing improved education could benefit health awareness. Poverty elimination and income generation programs among women are also likely to improve awareness about community health clinics in the target population. Special policy attention is required to address the regional variation of awareness about Community clinics.

## Introduction

While Bangladesh has achieved notable achievement in improving the health of the population, some health indicators still remain poor. One of the foremost factors contributing to this situation is the under-utilization of community clinic services (CCS). Reasons for under-utilization of CCS have been attributed to distance of the facility from home, lack of awareness on the value of services, perceived poor quality of care, cultural and social belief systems, discrimination against those of low socio-economic status and perceived high access costs [1–2].

In Bangladesh, rural dwellers are most-at-risk and vulnerable regarding health care facilities. The circumstance is worse for women when it comes to health care seeking behaviours and the care they receive during pregnancy and after childbirth. Health care seeking behaviour is not a secluded affair, but an integral part of a woman’s position in her society. It is a product of a progressive combination of her social, economic, household and religious components. The manner of seeking care can be too complex to be illustrated in a simple word. The choice to seek a specific care is a multifactorial outcome of her social forces, personal needs, the location of the health care services and the availability and qualifications of the care providers [1].

Several factors which have been known to determine health care seeking behaviours for safe motherhood among rural dwellers in Bangladesh include educational attainment, economic strength, age at marriage and location amongst others. The value of gynaecological and obstetric care in the development the society is known and made top priority. Without improving women’s health care seeking behaviour regarding antenatal, delivery and postnatal care, the total sustainable development goals of the country cannot be achieved [1].

Statistics has shown that large number of women in rural areas of Bangladesh encounter severe complications during pregnancy and childbirth. Sometimes, the women do not know about health care services existing for their use, while under-utilization of healthcare services could lead to maternal morbidity and mortality. Health care seeking behaviour model was used to investigate maternal health services utilization women of reproductive age in Bangladesh [3]. In the study, educational attainment became a prominent determinant for utilization of antenatal care, types of assistance at delivery and choice of place of delivery. Other key factors obtained were place of residence and household wealth index. More so, the place of delivery was vital in care seeking behaviour among women. Furthermore, there has been report of minute proportion of deliveries taking place in hospitals where better services are available. There are numerous complications including maternal morbidity and mortality occasioned by the place of delivery. Large proportion of deliveries has been found to take place at home. The deliveries were often aided by unskilled birth attendants in Bangladesh [4].

Delay in accessing gynaecological and obstetric care is also a major factor in women’s health. The delay is mainly associated to maternal morbidity and mortality in rural areas of Bangladesh [5]. The reasons identified are long waiting-time for results of treatment, inability to understand the severity of illnesses and a lack of financial support. Furthermore, socio-culturally identified gender roles put different limits and norms on women. However, gender issue imposes specific reproductive responsibilities on women, and hence results in early and excessive childbearing. In short, gender roles are also attributable to women’s lack of power to make decisions about their reproductive behaviour and to enhance their economic power to become self-reliant in decision-making [6].

Primary health care system is a vital tool for functional health care services in any society [7]. About 65% of people in Bangladesh are rural dwellers, while the majority of them are of low socioeconomic status [8]. A rural level facility such as the CCs becomes demanding to provide health care services to the most-at-risk and vulnerable women. Consequent upon this, the CCs system was developed to ameliorate the situation. But, less awareness is available to describe the importance of utilizing the CCS [9]. A key method to scrutinize the utilization of CCS could be by assessing the level of awareness of the women about the facilities.

In order to enhance the condition, specifically utilization and access the Ministry of Health and Family Welfare have built over 14,000 community clinics through the programme ‘Revitalisation of Community Health Care Initiative in Bangladesh (RCHCIB)—Community Clinic Project’ mainly to provide essential care package for women and children. But, these facilities are yet to gain full-scale level of utilization due to several factors [2]. Therefore, it is paramount to examine health outcomes using social and economic factors [10–11]. Utilization of healthcare services is crucial and interventions that improve the use will also improve the general health outcomes [12]. Nevertheless, rural women should have adequate awareness about community health services [13].

The aim of this study was to evaluate the associations of socioeconomic determinants with awareness and utilization of Community Clinic Services among women of rural dwellers in Bangladesh.

## Materials and methods

### Data sources

The study design used was a cross-sectional study based on Bangladesh Demographic and Health Survey (BDHS)-2014. The study utilized information from secondary data set in the survey conducted by the National Institute of Population Research and Training of Bangladesh. Furthermore, Mitra and Associates, a Bangladesh-based research firm, conducted the survey. ICF International of Calverton, USA, provided technical assistance for the survey and the U.S. Agency for International Development (USAID) provided financial support for the survey. The 2014 BDHS was a nationally representative data set. The survey used a sampling frame from the list of enumeration areas (EAs) of the 2011 Population and Housing Census of the People’s Republic of Bangladesh, provided by the Bangladesh Bureau of Statistics (BBS). The primary sampling unit (PSU) for the survey was an EA created to have an average of about 120 households.

The Demographic and Health Surveys (DHSs) are free, public datasets, though researchers must register with MEASURE DHS and submit a request before access to DHS data is granted. This data request system ensures that all users understand and agree to basic data usage ethics standards.

### Study setting and sampling details

There are seven administrative divisions in Bangladesh—Dhaka, Rajshahi, Rangpur, Chittagong, Khulna, Barisal and Sylhet. One division is subdivided into districts (zilas), and each district is divided into administrative units (upazilas), which are further divided into urban and rural areas. A two-stage stratified sample of households was the basis for this survey. In the first stage, 207 clusters in urban areas and 393 in rural areas were selected total 600 enumeration areas with proportional probability. In the second stage, on average, 30 households were selected based on the demographic and health survey variables, for both the urban and rural areas in seven divisions by systematic sample. The survey selected 17,842 residential households within this study design [14].

### Data extraction

The outcome variables of this study include the awareness and utilization of women regarding community clinic services. Also, the types of services provided by community clinic were also considered as outcomes. Several questions were used to measure awareness, utilization and types of services received from the community clinic in the BDHS-2014. In the survey, women were asked questions such as; if they were aware of any community clinic in their area; among those who answered affirmative, they were asked if they visit the community clinic in the past three months. Again, women who had visited were asked the type of services they received. The independent variables of the study were socio-demographic variables such as; age, educational attainment, wealth index, place of residence or geographical division, frequency of reading newspaper, listening to radio, watching television, sex of household head.

### Data analysis

Percentage was used to report categorical variables for univariate analysis method. In the bivariate technique, Chi-squared test was used to measure the association between awareness of community clinic services and socio-demographic variables. Binary logistic regression was used to calculate odds ratio (OR) and 95% confidence interval (CI) to examine the associations between awareness and background characteristics. The Statistical Package for Social Science (SPSS) version 22.0 (SPSS Inc., Chicago, IL, USA) was used for all analyses. All tests were two-sided, and statistical significance was considered at p < 0.05.

### Ethical clearance

The demographic and health survey program has standard methods for maintaining the confidentiality of participants. Before each interview, the interviewer is made to read the consent statement and inform the participant of voluntary participation and that she or he has the freewill to terminate the interview at any stage of the process. More so, the ICF International certifies that the survey complies with the United States Department of Health and Human Services rules for the protection of participants and ensures that the survey follows the laws and regulations of the nation. Hence, approval for this study was not needed since the data is available in the public domain.

## Results

In total 11,673 women were included in the present study. Table 1 illustrates that more than half of the participants (52%) were below 30 years of age. Highest participation was from Chittagong division (15.7%) with Barisal accounting for lowest percentage of participation (Barisal). More than a quarter of the women (28.1%) had no experience of formal education, about one-third had primary schooling (31.3%), and two-fifth (40.7%) had secondary or above qualification. A vast majority of the women (88.6%) were from male-headed households. Regarding household economic status, about half of the participants were from poorest (23.3%) to poorer (25.2%) wealth groups, and only 9.6% were from the wealthiest group. Regarding paper and electronic media use, 89.7% of the women reported almost never reading newspaper or magazines, 4.2% reported listening to radio and 49.0% of watching TV occasionally. Results of the cross-tabulation shows that women who were aware of community clinic were more likely to be aging below 30 years, being located in Rangpur district, having higher educational achievement, living in male-headed and richer/richest households. Surprisingly, women who reported not utilizing newspaper, radio and TV were more likely to be aware of community clinics.

**Table 1:** Baseline characteristics of the study population (n = 11,673), BDHS 2014.

Variable	N (%)	Aware of community clinic		p-value
		Yes (36.7)	No (63.3)	
**Age**				0.748
15–29 Years	6066 (52.0)	2232 (52.2)	3834 (51.9)	
30–49 Years	5607 (48.0)	2047 (47.8)	3560 (48.1)	
**Division**				<0.001***
Barisal	1455 (12.5)	751 (17.6)	704 (9.50)	
Chittagong	1838 (15.7)	617 (14.4)	1221 (16.5)	
Dhaka	1738 (14.9)	469 (11.0)	1269 (17.2)	
Khulna	1700 (14.6)	645 (15.1)	1055 (14.3)	
Rajshahi	1667 (14.3)	586 (13.7)	1081 (14.6)	
Rangpur	1803 (15.4)	818 (19.1)	985 (13.30)	
Sylhet	1472 (12.6)	393 (9.20)	1079 (14.6)	
**Educational attainment**				<0.001***
Nil	3052 (26.1)	977 (22.8)	2075 (28.1)	
Primary	3646 (31.2)	1334 (31.2)	2312 (31.3)	
Secondary/higher	4975 (42.6)	1968 (46.0)	3007 (40.7)	
**Sex of household head**				0.069
Male	10342 (88.6)	3761 (87.9)	6581 (89.0)	
Female	1331 (11.4)	518 (12.1)	813 (11.0)	
**Wealth index**				0.033***
Poorest	2725 (23.3)	440 (10.3)	677 (9.20)	
Poorer	2939 (25.2)	754 (17.6)	1359 (18.4)	
Middle	2779 (23.8)	1041 (24.3)	1738 (23.5)	
Richer	2113 (18.1)	1099 (25.7)	1840 (24.9)	
Richest	1117 (9.60)	945 (22.1)	1780 (24.1)	
**Frequency of reading newspaper/magazine**				0.010***
Almost never	10467(89.7)	3789 (88.5)	6678 (90.3)	
Sometimes	1206 (10.3)	490 (11.5)	716 (9.70)	
**Frequency of listening to radio**				0.249
Almost never	11185(95.8)	4083 (95.4)	7102 (96.1)	
Sometimes	488 (4.20)	196 (4.60)	292 (3.90)	
**Frequency of watching TV**				0.474
Almost never	5953 (51.0)	2164 (50.6)	3789 (51.2)	
Sometimes	5720 (49.0)	2115 (49.4)	3605 (48.8)	

***p significant at 0.05 for *χ*^2^-test.

### Pattern of utilization of community clinic services

Table 2 shows the types and relative degree of the services received from community clinics vary substantially across divisions. It also reveals that most of the participants attended clinics to seek healthcare services for children. Highest percentage of attendance for family planning and antenatal care were observed in Rangpur, immunization, tetanus, vitamin-A capsule and child growth monitoring in Khulna.

**Table 2:** Division-wise proportion usage of community clinic services, BDHS 2014.

Division	FP	Immunization	Tetanus	ANC	Vitamin-A for children	General healthcare of children	Child growth monitoring
**Barisal**	14.1	14.5	24.2	19.5	11.4	12.5	20.0
**Chittagong**	13.1	20.0	12.1	9.80	17.1	11.8	-
**Dhaka**	8.60	8.20	12.1	7.30	-	9.50	-
**Khulna**	23.4	20.9	30.3	17.1	45.7	17.8	40.0
**Rajshahi**	10.3	14.5	12.1	14.6	2.90	16.0	-
**Rangpur**	27.5	10.0	6.10	29.3	17.1	24.1	10.0
**Sylhet**	3.10	11.8	3.0	2.40	5.70	8.30	30.0

Notes: FP = Family planning, ANC = Antenatal care,

### Factors of association between community clinic awareness and explanatory variables

Results of multivariate logistic regression are shown in Table 3. Here, women from Barisal division are more likely to be aware of community clinic services when compared to other divisions. In addition, increase in economic status increases the level of awareness of community clinic services among women in rural areas. Furthermore, results showed a pattern of increasing level of awareness by increase in educational attainment of women. Finally, reading newspaper was a significant predictor of awareness of community clinic services.

**Table 3:** Factors associated with awareness of community clinic among women in Bangladesh, BDHS 2014.

Variable	*p*-value	AOR (95%CI)	*p*-value	COR (95%CI)
**Division**				
Barisal (ref)		1.0		
Chittagong	<0.001*	0.452 (0.391–0.522)	<0.001*	0.474 (0.411–0.546)
Dhaka	<0.001*	0.338 (0.291–0.393)	<0.001*	0.346 (0.299–0.402)
Khulna	<0.001*	0.560 (0.485–0.647)	<0.001*	0.573 (0.497–0.661)
Rajshahi	<0.001*	0.510 (0.441–0.589)	<0.001*	0.508 (0.440–0.587)
Rangpur	0.002*	0.797 (0.693–0.917)	<0.001*	0.778 (0.678–0.894)
Sylhet	<0.001*	0.341 (0.292–0.399)	<0.001*	0.341 (0.293–0.399)
**Wealth index**				
Poorest (ref)		1.0		
Poorer	0.178	1.080 (0.966–1.208)	0.034*	1.125 (1.009–1.254)
Middle	0.125	1.096 (0.975–1.231)	0.032*	1.128 (1.011–1.260)
Richer	0.191	1.090 (0.958–1.241)	0.468	1.045 (0.928–1.177)
Richest	<0.001*	1.337 (1.137–1.573)	0.006*	1.224 (1.060–1.413)
**Educational attainment**				
No education (ref)		1.0		
Primary	0.009*	1.149 (1.035–1.276)	<0.001*	1.225 (1.107–1.357)
Secondary	<0.001*	1.251 (1.123–1.392)	<0.001*	1.370 (1.242–1.510)
Tertiary	0.017*	1.269 (1.043–1.543)	<0.001*	1.526 (1.286–1.809)
**Frequency of reading newspaper**				
Not at all (ref)		1.0		
Less than once a week	0.127	1.128 (0.966–1.317)	0.009*	1.209 (1.048–1.394)
At least once a week	0.839	1.024 (0.812–1.293)	0.096	1.200 (0.968–1.487)

AOR = Adjusted odds ratio; ref = reference category.

*significant at p<0.05.

P-values are from the test score

## Discussion

This study reported poor awareness and utilization of Community Clinic Services (CCS) among women of reproductive age in rural Bangladesh. This is similar to the findings of studies on health care seeking behaviours in other parts of the world such as USA, Nepal and Nigeria [15–18]. Several latent factors could be responsible for the low prevalence of awareness and utilization of CCS such as inaccessibility to healthcare facilities and lack of health education system could be key obstacles to ensuring optimum healthcare services most-at-risk and vulnerable women living in rural areas. In addition, poor knowledge about the signs and symptoms of illnesses, stigmatization and lack of confidentiality could also be attributable. Religious and cultural beliefs could be additional reasons for poor utilization among rural dwellers in Bangladesh. Furthermore, attitudes toward health awareness and treatment-seeking behaviour are consequent upon the level of motivation to employ suitable health care services [19–20].

In order to enhance utilization of community clinic services, the media can help to improve the level of awareness, good health education system and improved accessibility in the hard-to-reach rural areas would make community leaders more actively involved in health care intervention programmes that target improving women’s behaviours towards health care, patient service-providers relationships, and the implementation of unique beneficiary packages for women attending antenatal clinic [21–22].

Prominent services reported in this study include family planning, immunization, tetanus, antenatal care, vitamin A, and general healthcare for children and child growth monitoring. Several divisions had no such services like child growth monitoring; also the prevalence of rural women attending antenatal care was low across geographical divisions [2,4]. In general, Khulna division took a lead in the services reported. Results from bivariate analysis showed statistical association between geographical/geopolitical division, wealth index, educational attainment, frequency of reading newspaper and awareness community clinic services. The results of our study indicated that Barisal division were most likely to awareness of community clinic services than six divisions. In addition, women of higher economic status were more likely to be aware of community clinic services. There was a “dose-response” type of association, which showed that awareness of CCS increases as the educational attainment increases [6,23]. Rural women with primary educational attainment were more likely to have awareness of CCS than those who did have formal education. This is in line with several studies involving health care utilization [24–29]. Educated women could have better opportunity to health information. Similarly, they are better equipped for initiating and controlling decision- making affairs regarding health issues [30]. Education is regarded as a prominent socioeconomic factor of health related attitude. Also, the frequency of reading newspaper also had significant association with awareness of CCS.

This showed socioeconomic attribute of individuals [31]. Where rural women of higher educational attainment become superior in the use of health information and services [32] and have better healthcare seeking behaviours. Local media could also provide the platform to health education for complete utilization of community clinic services.

## Strength and limitations

The study involved a nationally representative and large data set with high response rate. However, the study has a drawback in that it utilized cross-sectional dataset and unable to establish causality.

## Conclusion

Poor awareness of community clinic services was dominant among rural women in Bangladesh. Education, economic status, geopolitical/geographical division and frequency of reading newspaper were significantly associated with CCS awareness among rural dwellers in Bangladesh. Communal effort healthcare providers and local leaders is needed to increase awareness and improved access of healthcare services through community clinic services. Stakeholders in health should provide sufficient mechanisms to support awareness and utilization health facilities in rural areas in Bangladesh.

## References

[1] Aktar S. Health Care Seeking Behaviour for Safe Motherhood: Findings from Rural Bangladesh. Bangladesh e-Journal of Sociology. 2012; 9(2), 57–70

[2] COMDIS-HSD. Research and development for effective service delivery. Improving primary health care using community clinics in rural Bangladesh. 2014. Project brief.

[3] Haque M. Individual's characteristics affecting maternal health services utilization: Married adolescents and their use of maternal health services in Bangladesh. Internet Journal of Health, 2009; 8(2), p. 16.

[4] Islam M., Chowdhury R. and Akhter H. Complications during pregnancy, delivery, and postnatal stages and place of delivery in rural Bangladesh. Health Care for Women International, 2006; 27(9), 807–821. doi: 10.1080/07399330600880368 17060180 10.1080/07399330600880368

[5] Killewo J., Anwar I., Bashir I., Yunus M. and Chakraborty J. Perceived delay in healthcare-seeking for episodes of serious illness and its implications for safe motherhood interventions in rural Bangladesh. Journal of Health, Population, and Nutrition, 2006; 24(4), 403–412. 17591337 PMC3001144

[6] Ahmed S. M., Adams A.M. Chowdhury M. and Bhuiya A. Gender, socioeconomic development and health-seeking behaviour in Bangladesh. Social Science and Medicine, 2000; 51(3), 361–371. 10855923 10.1016/s0277-9536(99)00461-x

[7] Starfield B. Is primary care essential? Lancet. 1994;344(8930):1129–33. 7934497 10.1016/s0140-6736(94)90634-3

[8] Alim MA, Sarker M, Selim S, Karim MR, Yoshida Y, Hamajima N. Respiratory involvements among women exposed to the smoke of traditional biomass fuel and gas fuel in a district of Bangladesh. Environ Health Prev Med. 2014;19(2):126–34. doi: 10.1007/s12199-013-0364-4 24105352 10.1007/s12199-013-0364-4 PMC3944038

[9] Normand C, Iftekar MH, Rahman SA. Assessment of the community clinics: effects on service delivery, quality and utilization of services. 2012.

[10] Marmot M. Social determinants of health inequalities. Lancet. 2005;365(9464):1099–104. doi: 10.1016/S0140-6736(05)71146-6 15781105 10.1016/S0140-6736(05)71146-6

[11] Benova L, Grundy E, Ploubidis GB. Socioeconomic position and health-seeking behaviour for hearing loss among older adults in England. J Gerontol B Psychol Sci Soc Sci. 2014; doi: 10.1093/geronb/gbu024 24663332 10.1093/geronb/gbu024 PMC4501830

[12] Mackenbach JP, Stirbu I, Roskam AJ, Schaap MM, Menvielle G, Leinsalu M, Kunst AE. Socioeconomic inequalities in health in 22 European countries. N Engl J Med. 2008;358(23):2468–81. doi: 10.1056/NEJMsa0707519 18525043 10.1056/NEJMsa0707519

[13] Carlton EL, Simmons L. Health decision-making among rural women: physician access and prescription adherence. Rural Remote Health. 2011;11:1599.

[14] The DHS Program. Data set access approval process. 2014. https://dhsprogram.com/data/Access-Instructions.cfm. Accessed 12 December 2016

[15] Spleen AM, Lengerich EJ, Camacho FT, Vanderpool RC. Health care avoidance among rural populations: results from a nationally representative survey. J Rural Health. 2014;30(1):79–88. doi: 10.1111/jrh.12032 24383487 10.1111/jrh.12032 PMC3882339

[16] Sreeramareddy CT, Shankar RP, Sreekumaran BV, Subba SH, Joshi HS, Ramachandran U. Care seeking behaviour for childhood illness–a questionnaire survey in western Nepal. BMC Int Health Hum Rights. 2006;6:7 doi: 10.1186/1472-698X-6-7 16719911 10.1186/1472-698X-6-7 PMC1543657

[17] Ogunlesi TA, Olanrewaju DM. Socio-demographic factors and appropriate health care seeking behavior for childhood illnesses. J Trop Pediatr. 2010;56(6):379–85. doi: 10.1093/tropej/fmq009 20167633 10.1093/tropej/fmq009

[18] Fan L, Shah MN, Veazie PJ, Friedman B. Factors associated with emergency department use among the rural elderly. J Rural Health. 2011;27(1):39–49. doi: 10.1111/j.1748-0361.2010.00313.x 21204971 10.1111/j.1748-0361.2010.00313.x PMC3058487

[19] Javali R, Wantamutte A, Mallapur MD. Socio-demographic factors influencing utilization of antenatal health care services in a rural area—a cross-sectional study. Int J Med Sci Public Health. 2014;3(3):308–12.

[20] Rashid SF, Akram O, Standing H. The sexual and reproductive health care market in Bangladesh: where do poor women go? Reprod Health Matters. 2011;19(37):21–31. doi: 10.1016/S0968-8080(11)37551-9 21555083 10.1016/S0968-8080(11)37551-9

[21] Stone L. Primary health care for whom? Village perspectives from Nepal. Soc Sci Med. 1986;22(3):293–302. 3961544 10.1016/0277-9536(86)90125-5

[22] Ahmed S, Khan MM. Is demand-side financing equity enhancing? Lessons from a maternal health voucher scheme in Bangladesh. Soc Sci Med. 2011;72(10):1704–10. doi: 10.1016/j.socscimed.2011.03.031 21546145 10.1016/j.socscimed.2011.03.031

[23] Bazzano AN, Kirkwood BR, Tawiah-Agyemang C, Owusu-Agyei S, Adongo PB. Beyond symptom recognition: care-seeking for ill newborns in rural Ghana. Trop Med Int Health. 2008;13(1):123–8. doi: 10.1111/j.1365-3156.2007.01981.x 18291010 10.1111/j.1365-3156.2007.01981.x

[24] Simsek H, Doganay S, Budak R, Ucku R. Relationship of socioeconomic status with health behaviours and self-perceived health in the elderly: a community-based study. Turkey: Geriatr Gerontol Int; 2013. 10.1111/ggi.12166 24118995

[25] Mishra SK, Mukhopadhyay S. Socioeconomic correlates of reproductive morbidity among adolescent girls in Sikkim, India. Asia Pac J Public Health. 2012;24(1):136–50. doi: 10.1177/1010539510375842 21266396 10.1177/1010539510375842

[26] Tsegay Y, Gebrehiwot T, Goicolea I, Edin K, Lemma H, Sebastian MS. Determinants of antenatal and delivery care utilization in Tigray region, Ethiopia: a cross-sectional study. Int J Equity Health. 2013;12:30 doi: 10.1186/1475-9276-12-30 23672203 10.1186/1475-9276-12-30 PMC3658893

[27] Ogunlesi TA, Ogunlesi FB. Family socio-demographic factors and maternal obstetric factors influencing appropriate health-care seeking behaviours for newborn jaundice in Sagamu, Nigeria. Matern Child Health J. 2012;16(3):677–84. doi: 10.1007/s10995-011-0765-1 21365297 10.1007/s10995-011-0765-1

[28] Ogunlesi TA. Maternal socio-demographic factors influencing the initiation and exclusivity of breastfeeding in a Nigerian semi-urban setting. Matern Child Health J. 2010;14(3):459–65. doi: 10.1007/s10995-008-0440-3 19156508 10.1007/s10995-008-0440-3

[29] Wamani H, Astrom AN, Peterson S, Tylleskar T, Tumwine JK. Infant and young child feeding in western Uganda: knowledge, practices and socioeconomic correlates. J Trop Pediatr. 2005;51(6):356–61. doi: 10.1093/tropej/fmi048 15947011 10.1093/tropej/fmi048

[30] Saito K, Korzenik JR, Jekel JF, Bhattacharji S. A case-control study of maternal knowledge of malnutrition and health-care-seeking attitudes in rural South India. Yale J Biol Med. 1997;70(2):149–60. 9493847 PMC2589065

[31] Kempen GI, Brilman EI, Ranchor AV, Ormel J. Morbidity and quality of life and the moderating effects of level of education in the elderly. Soc Sci Med. 1999;49(1):143–9. 10414847 10.1016/s0277-9536(99)00129-x

[32] Ogunlesi TA, Okeniyi JAO, Oyedeji GA, Oyedeji OA. The influence of maternal socioeconomic status on the management of malaria in their children: implications for the ‘Roll Back Malaria’ initiative. Niger J Paediatr. 2005;32:2

